# Association of circulating monocyte number and monocyte–lymphocyte ratio with cardiovascular disease in patients with bipolar disorder

**DOI:** 10.1186/s12888-024-06105-3

**Published:** 2024-10-11

**Authors:** Pao-Huan Chen, Chi-Kang Chang, Yen-Kuang Lin, Shuo-Ju Chiang, Nguyen Ngoc Trang

**Affiliations:** 1 Department of Psychiatry, Taipei Medical University Hospital, Taipei, Taiwan; 2 Psychiatric Research Center, Taipei Medical University Hospital, Taipei, Taiwan; 3 Department of Psychiatry, School of Medicine, College of Medicine, Taipei Medical University, Taipei, Taiwan; 4 Department of Psychiatry, Taipei City Psychiatric Center, Taipei City Hospital, Taipei, Taiwan; 5 Graduate Institute of Athletics and Coaching Science, National Taiwan Sport University, Taoyuan, Taiwan; 6 Division of Cardiology, Department of Internal Medicine, Taipei City Hospital Yangming Branch, Taipei, Taiwan; 7 School of Biomedical Engineering, College of Biomedical Engineering, Taipei Medical University, Taipei, Taiwan; 8 Radiology Center, Bach Mai Hospital, Hanoi, Vietnam

**Keywords:** Mania, Cardiovascular disease, Leukocyte, Monocyte– lymphocyte ratio, Neutrophil–lymphocyte ratio, Platelet–lymphocyte ratio, Inflammation, Uric acid

## Abstract

**Background:**

Cardiovascular disease (CVD) is the leading cause of excessive and premature mortality in patients with bipolar disorder (BD). Despite immune cells participating considerably in the pathogenesis of CVD, limited data are available regarding leukocyte phenotypes in patients with BD and CVD. This study aimed to evaluate associations between circulating leukocyte subset and CVD among patients with BD.

**Methods:**

A total of 109 patients with BD-I and cardiologist-confirmed CVD diagnosis (i.e., case) were matched with 109 BD-I patients without CVD (i.e., control) according to the age (± 2 years), sex, and date of most recent psychiatric admission because of acute mood episode (± 2 years). Leukocyte subset data were retrieved from complete blood count tests performed on the next morning after the most recent acute psychiatric admission.

**Results:**

During the most recent acute psychiatric hospitalization, circulating monocyte counts in the case group were significantly higher than those in the age- and sex-matched controls (*p* = 0.020). In addition, monocyte–lymphocyte ratios (MLRs) in the case group were significantly higher than those in the control group (*p* = 0.032). Multiple logistic regression showed that together with serum levels of uric acid and manic symptoms, circulating monocyte counts (95% CI, OR: 1.01–1.05) and MLRs (95% CI, OR: 1.01–1.09) were significantly associated with CVD in patients with BD, respectively.

**Conclusions:**

Monocyte activation in an acute manic episode may play a critical role in the pathogenesis of CVD among patients with BD. Future research is required to investigate markers of monocyte activation and indices of cardiovascular structure and function across the different mood states of BD.

## Background

Bipolar disorder (BD) is a serious mental illness that often has multifaceted manifestations [1, 2]. Beyond the mood symptoms and cognitive dysfunction, patients with BD also experience an increased risk of medical morbidity and mortality across the multiple organ systems [3–5]. Systemic inflammation is proposed as an underlying mechanism driving the multisystem manifestation of BD [6].

International studies have consistently reported that, among the various organ systems, cardiovascular disease (CVD) is the leading cause of excessive and premature death in the population with BD [7, 8]. Of particular note, one of the key mechanisms involved in the pathogenesis of CVD is the inflammation [9, 10]. Biomarkers related to the inflammatory process of CVD, such as the leukocyte count, C-reactive protein, cytokines, and oxidative stress, are significantly associated with CVD risk and outcomes in both general population and individuals with mood disorders [11–13]. Above all, leukocyte count and its subtypes have been afforded considerable attentions because of their critical roles in the pathogenic mechanisms of endothelial dysfunction, blood pressure dysregulation, and atherosclerotic plaque formation [9, 10, 14]. Furthermore, the particular leukocyte subsets, such as higher neutrophil to lymphocyte ratio (NLR), monocyte to lymphocyte ratio (MLR), and platelet to lymphocyte ratio (PLR), have been reported to be associated with BD [15–18]. With the characteristics that leukocyte subset derived from a complete blood count has the advantage of easy accessibility, acceptable reproducibility, and inexpensiveness [19], leukocyte count may become a valuable biomarker in the prediction of CVD risk at an individual level of patients with BD. However, to date limited data are available regarding leukocyte subsets in patients with BD comorbid with CVD. Such insight may guide future research into cellular biomarkers and novel treatments to reduce the high CVD mortality in the BD population.

This study aimed to evaluate the associations between a circulating leukocyte subset and CVD among patients with BD. We focused on ischemic heart disease and hypertension because these are the principle vascular diseases in individuals with BD [20, 21] and immune cells play an essential in the genesis of these two types of vascular diseases [9, 10, 22]. We hypothesized that certain blood leukocyte subpopulations were associated with the increased risk of ischemic heart disease and hypertension in patients with BD. We did not hypothesize a particular leukocyte subset in the associations because of the exploratory nature of the present study.

## Methods

### Study sample

Data used for analyses in this study were retrieved from the database described in our previous research [23, 24]. To explore the potential roles of leukocytes in the risk prediction of CVD among patients with BD, we focused on blood cell counts, which were not examined in our prior research. The study protocol was approved by the institutional review boards of Taipei Medical University Hospital (Protocol Number: 201312054) and Taipei City Psychiatric Center (Protocol Number: TCHIRB-1030105), and waived the need for informed consent due to the retrospective chart review and secondary analyses of de-identified data under the regulation (code: 1010265083) of Ministry of Health and Welfare in Taiwan (https://dep.mohw.gov.tw/DOMA/fp-2782-9538-106.html).

**Fig. 1:**
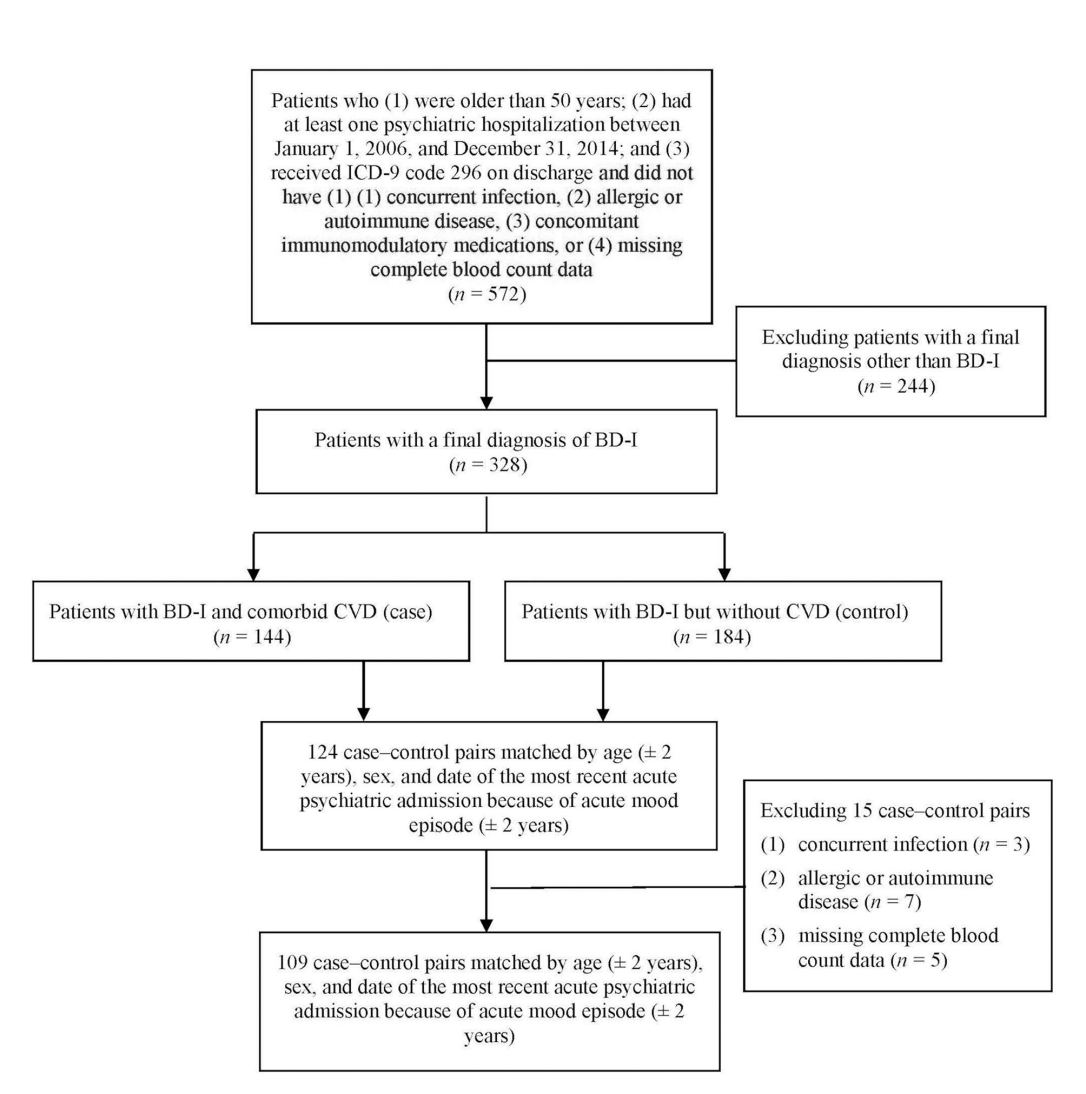
Study flow diagram. Abbreviations: BD-I = bipolar I disorder, CVD = cardiovascular disease, ICD-9 = International Statistical Classification of Diseases and Related Health Problems, Ninth Revision

The sampling procedures employed have been described elsewhere [23, 24] and are thus outlined only briefly herein, with a focus on aspects relevant to the current analyses (Fig. 1). Using the data files from Taipei Medical University Hospital and Taipei City Psychiatric Center, we identified potential patients (*n* = 572) according to the following inclusion criteria: (1) older than 50 years; (2) had at least one psychiatric admission to Taipei Medical University Hospital and Taipei City Psychiatric Center between January 1, 2006, and December 31, 2014; and (3) received code 296 in the *International Statistical Classification of Diseases and Related Health Problems*,* Ninth Revision* (ICD-9) on discharge. The exclusion criteria were the following conditions: (1) concurrent infection, (2) allergic or autoimmune disease, (3) concomitant immunomodulatory medications, and (4) missing complete blood count data. After comprehensive chart review by two board-certified psychiatrists involved in this study (PHC and CKC), two hundreds and forty-four patients with a final diagnosis other than BD-I were excluded. Among the remaining 328 patients with BD-I, 144 were determined to have comorbid CVD. The criteria for a diagnosis of CVD were: (1) a definitive diagnosis of CVD on the discharge note; (2) standard treatment for CVD for at least 6 months; or (3) significant physical or laboratory findings that supported the diagnosis of CVD as determined by a board-certified cardiologist (SJC). Afterward, each patient in the case group (i.e., with BD-I and comorbid with CVD) was matched with one patient with BD-I but without CVD as a control according to their age (± 2 years), sex, and date of their most recent acute psychiatric admission because of acute mood episode (± 2 years). Among the 124 case–control pairs, 15 case–control pairs were excluded because of the following conditions: concurrent infection (*n* = 3), allergic or autoimmune disease (*n* = 7), and missing complete blood count data (*n* = 5). The remaining 109 case–control pairs were included in the present analyses.

### BD characteristics

Two board-certified psychiatrists retrospectively reviewed medical records using a case-note form at the Taipei Medical University Hospital and Taipei City Psychiatric Center. The case-note form has been used since 1980 and contains information regarding patient’s demographic background, clinical characteristics of psychiatric disorders, concurrent physical illness, and results of physical examinations and laboratory tests. To date, the case notes have provided data for numerous clinical studies published in the peer-reviewed journals [25–27].

Among the clinical variables related to BD characteristics, we defined an onset of BD as the occurrence of affective symptoms, either depression or mania, that caused impairment of a patient’s occupational and social function or resulted in psychiatric hospitalization. A psychotic feature was recorded if patients experienced hallucinations or delusions within any mood episode. Delusion or hallucination content consistent with a typical manic or depressive theme was referred to as a mood-congruent psychotic feature. The rapid-cycling feature was defined as at least four mood episodes within a 12-month period.

### Cardiometabolic risk factor

To capture cardiometabolic variables that potentially indicate CVD risk, we reviewed medical records and documented information regarding medical comorbidities and laboratory tests routinely performed on the next morning after the most recent acute psychiatric admission. We used the guidelines of the Health Promotion Administration in Taiwan [28] to determine the cut-off points for obesity, prediabetes, type II diabetes mellitus, hyperlipidemia and hyperuricemia. That used for obesity was a body mass index over 27 kg/m^2^. That used to define prediabetes and type II diabetes mellitus was a serum fasting glucose level between 125 mg/dL and 120 mg/dL and above 126 mg/dL, respectively. Those used to determine hyperlipidemia were a serum triglyceride level above 200 mg/dL, total cholesterol above 240 mg/dL, and low-density lipoprotein (LDL) cholesterol above 130 mg/dL. A serum uric acid level above 6.8 mg/dL indicated hyperuricemia. Patients who received treatment for prediabetes/type II diabetes mellitus, hyperlipidemia or gout/hyperuricemia were recorded as having these three metabolic diseases even if the results of their blood biochemistry tests were within the normal ranges.

### Blood leukocyte subset

We retrieved leukocyte subset data from the complete blood count test routinely performed the next morning after the most recent acute psychiatric admission. As a result of the test timing, the leukocyte subset in this study reflected the leukocyte distributions in acute mood episodes. We also analyzed platelet data in reference to the growing literature reporting that platelets are a type of inflammatory cell mediating pathogenesis of CVD [29]. In addition to the cell number analyses, we computed MLR, NLR, and PLR using the monocyte, neutrophil, or platelet count divided by lymphocyte count, respectively.

### Statistical analysis

Group differences in the continuous variables were examined utilizing an unpaired *t* test. In instances where the assumptions of normality were not met, Mann–Whitney U tests were applied. The normality assumption of variables was examined using the Shapiro-Wilk test. The chi-square test was employed to evaluate group differences in the categorical variables. Multiple logistic regressions were performed to predict the probability of a CVD event based on the significant (*p* < 0.05) independent variables in the univariate analyses. The logistic regression considers fitting an equation of the following outline to the data:

$$\log \,\left( {\frac{p}{{1 - p}}} \right) = {\beta _0} + {\beta _1}{X_1} + {\beta _2}{X_2} + \ldots {\beta _n}{X_n}$$

where p is the probability of CVD and ranges from 0 to 1, X_i_ are the independent variables to be selected, and β_i_ are the parameters of the logistic regression model.

All data analyses were performed using SAS 9.4 (SAS Institute, Cary, NC, USA). On account of the exploratory nature of this study, the univariate analyses were presented without Bonferroni corrections. A *p* value of < 0.05 was considered significant.

## Results

### Clinical characteristics

A total of 109 case–control pairs matched based on the age (± 2 years), sex, and date of most recent psychiatric admission because of acute mood episode (± 2 years) were analyzed in this study. The distributions of CVD in the case group confirmed by medical records and cardiologist’s diagnosis were as follows: ischemic heart disease (*n* = 29, 26.6%), hypertension (*n* = 64, 58.7%), and both ischemic heart disease and hypertension (*n* = 16, 14.7%). The mean age at CVD diagnosis was 61.9 ± 5.0 years, and that at the most recent acute psychiatric admission was 59.8 ± 5.5 years, approximately 2 years prior to CVD diagnosis (Table 1). Compared with the control patients (67.0%), a significantly high proportion of patients in the case group experienced mania during their most recent acute psychiatric hospitalization prior to CVD (84.4%, *p* = 0.003). No other clinical variables, including medications in most recent psychiatric hospitalization before CVD, differed significantly between the two groups.

**Table 1:** Clinical characteristics

	Case Group (*N* = 109)	Control Group (*N* = 109)	*p*
**Categorical variables**			
Men, n (%)	36 (33.0)	36 (33.0)	1.000
Cigarette smoking, n (%)	24 (22.0)	28 (25.7)	0.526
Lifetime suicide attempt, n (%)	55 (50.5)	45 (41.3)	0.175
Lifetime rapid-cycling feature, n (%)	19 (17.4)	23 (21.1)	0.493
Lifetime mood-congruent psychotic feature, n (%)	67 (61.5)	64 (58.7)	0.678
Lifetime mood-incongruent psychotic feature, n (%)	45 (41.3)	36 (33.0)	0.208
Most recent psychiatric hospitalization before CVD			
Mania, n (%)	92 (84.4)	73 (67.0)	0.003
Lithium, n (%)	33 (30.3)	44 (40.4)	0.120
Valproic acid, n (%)	64 (58.7)	66 (60.1)	0.783
SGA, n (%)	75 (68.8)	85 (78.0)	0.127
Antidepressant, n (%)	5 (4.6)	7 (6.4)	0.554
**Continuous variables**			
Age, years, mean (SD)	61.9 (5.0)	61.7 (5.0)	0.788
Age of onset, years, median (IQR)	31.0 (18.0)	31.0 (16.5)	0.719
Age at most recent psychiatric hospitalization, years, median (IQR)	59.0 (7.0)	59.0 (7.0)	0.964
Total psychiatric hospitalization, times, median (IQR)	8.0 (6.0)	7.0 (6.5)	0.231

Abbreviations: CVD = cardiovascular disease, IQR = interquartile range, SD, standard deviation, SGA = second-generation antipsychotics

### Medical comorbidities and metabolic profile

Table 2 lists the medical comorbidities and metabolic parameters that may confer the risk of CVD in patients with BD. Relative to the control group (obesity: 23.9%), a significantly higher proportion of patients in the case group had obesity (36.7%, *p* = 0.040). In addition, patients in the case group exhibited significantly higher mean body mass index values than those in the control group (*p* = 0.048). Regarding blood biochemistry test parameters during the most recent acute psychiatric hospitalization prior to CVD, the median levels of serum uric acid were significantly higher in the case group compared with those in the control (*p* = 0.019) although there were no significant differences in the diagnoses of gout/ hyperuricemia between the two groups. No significant difference was observed between the two groups in terms of mean serum levels of fasting glucose, triglycerides, total cholesterol, and LDL cholesterol.

**Table 2:** Medical comorbidities and metabolic parameters in the most recent acute psychiatric hospitalization before cardiovascular disease diagnosis

	Case Group (*N* = 109)	Control Group (*N* = 109)	*p*
**Categorical variables**			
Obesity, n (%)	40 (36.7)	26 (23.9)	0.040
Prediabetes/ type II diabetes mellitus, n (%)	46 (42.2)	42 (38.5)	0.679
Hyperlipidemia, n (%)	38 (34.9)	41 (37.6)	0.673
Gout/hyperuricemia, n (%)	14 (12.8)	17 (15.6)	0.437
**Continuous variables**			
Body mass index, kg/m^2^, mean (SD)	26.1 (4.3)	24.9 (4.0)	0.048
Fasting serum glucose, mg/dL, median (IQR)	101.0 (42.0)	97.0 (29.0)	0.273
Serum triglycerides, mg/dL, median (IQR)	113.0 (40.0)	111.0 (89.0)	0.788
Serum total cholesterol, mg/dL, median (IQR)	197.0 (46.0)	176.0 (49.0)	0.103
Serum LDL cholesterol, mg/dL, mean (SD)	115.5 (33.7)	114.0 (43.9)	0.871
Serum uric acid, mg/dL, median (IQR)	6.6 (2.4)	5.9 (1.9)	0.019

Abbreviation: IQR = interquartile range, LDL = low-density lipoprotein, SD = standard deviation

### Blood cell count and leukocyte subset

**Fig. 2:**
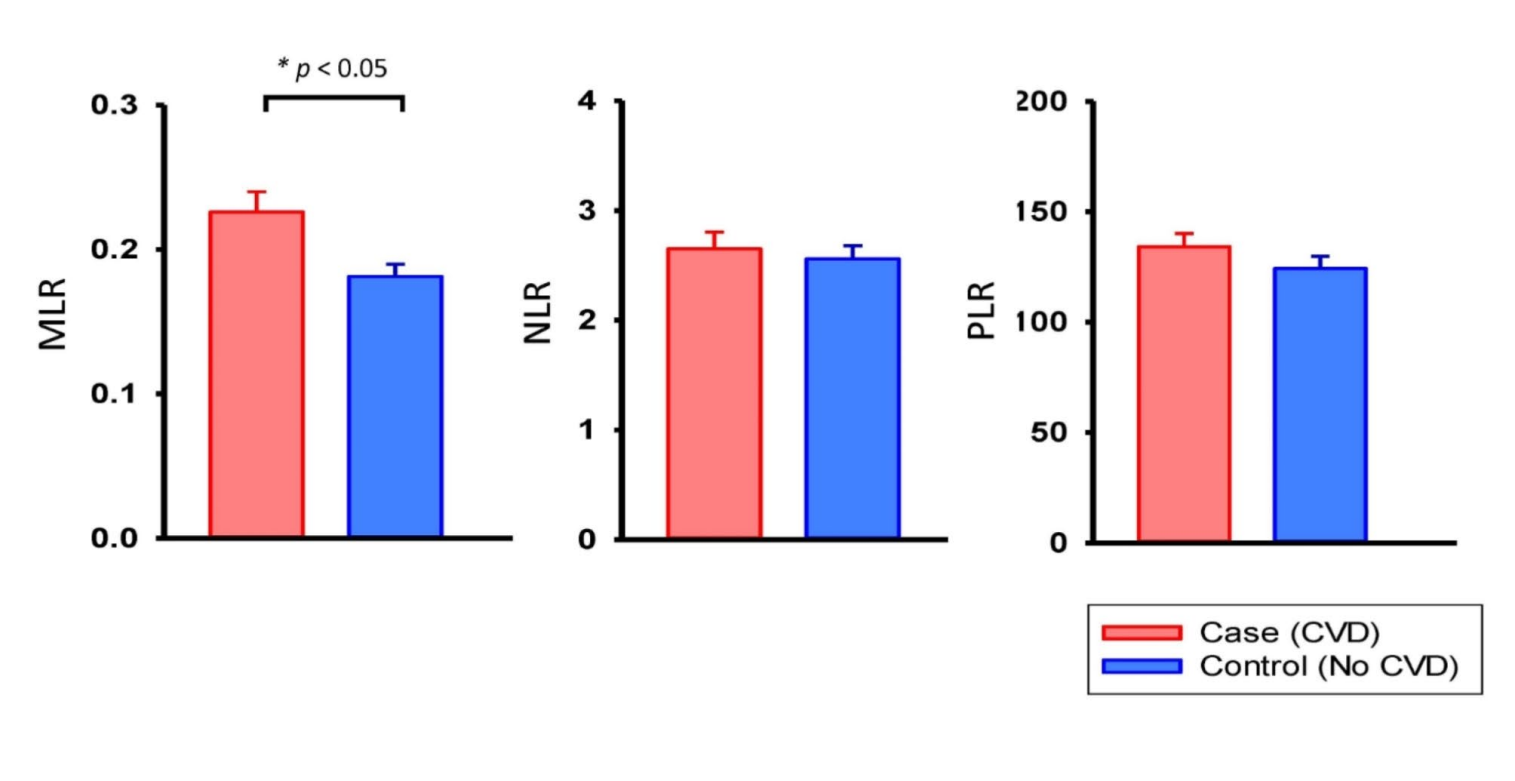
Monocyte–lymphocyte ratios (MLRs), neutrophil–lymphocyte ratios (NLRs) and platelet–lymphocyte ratios (PLRs) in the most recent acute psychiatric hospitalization before cardiovascular disease diagnosis

Table 3 presents the blood cell count and leukocyte subset during the most recent acute psychiatric hospitalization before CVD diagnosis. The median values of the monocyte count in the case group were significantly higher than those in the control group (*p* = 0.020). In addition, the median values of MLRs in the case group were also significantly higher than those in the control group (*p* = 0.032; Fig. 2). By contrast, no significant difference was observed in the number of leukocytes, neutrophils, lymphocytes, platelets, NLR, or PLR.

**Table 3:** Blood cell count and leukocyte subset in the most recent acute psychiatric hospitalization before cardiovascular disease diagnosis

	Case Group (*N* = 109)	Control Group (*N* = 109)	*p*
Leukocyte, 10^3^/µL, median (IQR)	6.94 (2.28)	6.82 (2.23)	0.782
Neutrophil, 10^3^/µL, median (IQR)	4.21 (2.64)	4.25 (1.76)	0.903
Lymphocyte, 10^3^/µL, median (IQR)	1.94 (0.88)	1.95 (0.83)	0.855
Monocyte, 10^3^/µL, median (IQR)	0.35 (0.20)	0.32 (0.17)	0.020
Platelet, 10^3^/µL, median (IQR)	235.0 (101.0)	224.0 (83.5)	0.318
Red blood cell, 10^6^/µL, mean (SD)	4.26 (0.57)	4.28 (0.47)	0.757

Abbreviation: IQR = interquartile range, SD = standard deviation

### Prediction models of CVD in BD

Table 4 details the results of the multiple logistic regressions based on the preliminary associations (*p* < 0.05) in the preceding analyses (Tables 1, 2 and 3). Blood monocyte count and MLR were separately applied to model 1 and model 2. According to model 1, the predictive validity of CVD was provided by the blood monocyte count (95% confidence interval [CI], odds ratio [OR]: 1.01–1.05), serum level of uric acid (95% CI, OR: 1.02–1.56), and manic symptoms (95% CI, OR: 1.07–5.69) in the most recent psychiatric hospitalization. The MLR in model 2 exhibited similar associations with CVD as the blood monocyte count in model 1, with the MLR (95% CI, OR: 1.01–1.09), serum level of uric acid (95% CI, OR: 1.01–1.55), and manic symptoms (95% CI, OR: 1.16–6.36) collectively providing the highest predictive validity of CVD in patients with BD.

**Table 4:** Logistic regression of factors in the most recent acute psychiatric hospitalization for cardiovascular disease diagnosis

Factors	Adjusted OR	95% CI for OR	*P*
**Model 1**			
Blood monocyte count	1.03	1.01–1.05	0.015
Serum uric acid level	1.26	1.02–1.56	0.033
Mania	2.47	1.07–5.69	0.034
Obesity	1.59	0.75–3.38	0.228
**Model 2**			
Blood MLR	1.05	1.01–1.09	0.012
Serum uric acid level	1.25	1.01–1.55	0.021
Mania	2.72	1.16–6.36	0.041
Obesity	1.70	0.80–3.63	0.169

Abbreviations: CI = confidence interval, MLR = monocyte–lymphocyte ratio, OR = odds ratio

## Discussion

To our knowledge, this is the first study to show that patients with BD comorbid with CVD exhibited higher monocyte numbers and MLRs in acute affective phase than those of the age- and sex-matched patients with BD and without CVD. In addition, the increased monocyte number and MLR during the acute mood episode were associated with CVD that occurred an average of 2 years later in patients with BD. The findings were in accordance with one recent report showing the associations between monocyte-activating cytokines and atherosclerosis in BD patients [30]. Contrary to studies on the general population [13, 31, 32], we observed no association of neutrophils and lymphocytes with CVD among patients with BD. The findings indicated a potentially distinct immune process driving pathogenesis of CVD in the general population and in those with BD.

In this study, we observed that circulating monocyte numbers and the MLR were both associated with CVD in patients with BD. The CVD diagnoses in this present sample were mainly consist of ischemic heart disease and hypertension. Mounting literature has indicated that among the different subsets of leukocytes, monocytes and monocyte-derived macrophages play a crucial role in the initiation and progression of coronary atherosclerotic plaque [9, 10]. Moreover, monocytes act on vasculature during the hypertension through the overactivation of renin–angiotensin system [33–35] and adverse vascular remodeling [22]. Thus far, the precise mechanisms underlying monocyte activation in the atherosclerogenesis and hypertension of BD still remain unclear. Considering that ischemic heart disease and hypertension are the leading vascular diseases in individuals with BD [20, 21], further mechanistic studies are needed in this area.

Manic symptoms were among the risk factors for CVD in our patients with BD. This finding is consistent with early studies that demonstrated associations between manic symptom burden and CVD in the BD population [36, 37]. Accumulating evidence has indicated that the pathophysiology of BD involves both neuroinflammatory and systemic inflammatory processes [38, 39]. In central nervous system, microglia activation produces proinflammatory cytokines and chemokines, leading to the dysregulation of neurotransmitters and neurocircuits related to mood regulation [40]. In peripheral circulation, monocytes are activated by the overexpression of monocyte- and macrophage-related chemokines during BD manic episodes [39]. In our current analyses, circulating monocyte numbers or the MLR together with mania raised the risk of CVD in patients with BD. These results suggested that monocytes were the major leukocytes linking the mood symptoms of BD to CVD. However, previous studies also indicated that depressive symptoms of BD also increase the risk of CVD [41, 42]. Furthermore, immunological markers ad leukocyte subsets during the depressive episodes are distinct from those in mania [43, 44]. Because the sample size for patients having depressive episodes in this study (case group: *n* = 17, control group: *n* = 36) are small, future studies with sufficient power are warranted to evaluate the associations between leukocyte subsets in bipolar depression and CVD.

Uric acid is the end product of purine catabolism. One meta-analysis has indicated that uric acid levels are elevated in patients with BD, especially during the manic or mixed phase [45]. Moreover, levels of uric acid together with inflammation correlate with aggressive behaviors in patients with BD [46]. These findings lead to a contention that purinergic system dysfunction is a potential pathophysiology of BD. It is worth noting that studies also reported the association of hyperuricemia with hypertension and coronary artery disease [47–49]. Mechanisms underlying these associations may involve monocyte activation, where uric acid upregulates nuclear factor kappa-light-chain-enhancer of activated B cells and nucleotide-binding oligomerization domain protein 3 inflammasomes [50]. In this study, we found that monocyte numbers or the MLR together with uric acid levels increased the CVD risk among patients with BD. The findings suggested future studies to evaluate whether monocytes interact with purinergic system leading to the genesis of CVD in BD.

The strength of the current findings is that the blood leukocyte counts were derived from routine tests in the hospital and were therefore valid and reliable. In addition, we matched cases and controls according to the age, sex, and date of most recent psychiatric admission, which reduced the possibility of confounding by age, sex, or chronological bias. However, several methodological shortcomings must be addressed when interpreting our findings. First, although this study analyzed data in the most recent acute psychiatric hospitalization approximately 2 years prior to the CVD diagnosis, we still could not rule out a possibility that some of our patients had already developed CVD but not been diagnosed before the admission. Second, sample size was not estimated a-priori. The matched case–control design may lead to a reduction in sample size and statistical power, potentially limiting our detection of differences in certain variables between the case and control groups. Third, to ascertain the validity of clinical diagnoses and obtain comprehensive data from medical records, we required patients to have had at least one hospitalization. The criteria may lead to the recruitment of patients with a more severe BD. Thus, our results may not be generalizable to those patients without hospitalization. Fourth, we were unable to assess all types of medications received by patients across the entire lifespan. In addition, we could not accurately evaluate adherence to medications based on the chart review method. Therefore, we could not exclude the confounding effects of medication exposure on the associations between monocytes and CVD risk.

## Conclusions

This study indicated that monocyte activation during the acute manic episode may play an essential role in the pathogenesis of ischemic heart disease and hypertension in patients with BD. To elucidate whether monocyte activation is correlated with the impaired vascular structure and function in patients with BD, future studies should evaluate monocyte activation markers and vascular measures across the various mood states.

## Data Availability

No datasets were generated or analysed during the current study.
